# Protein targets of thiazolidinone derivatives in *Toxoplasma gondii* and insights into their binding to ROP18

**DOI:** 10.1186/s12864-018-5223-7

**Published:** 2018-11-29

**Authors:** Diego Molina, Rodrigo Cossio-Pérez, Cristian Rocha-Roa, Lina Pedraza, Edwar Cortes, Alejandro Hernández, Jorge E. Gómez-Marín

**Affiliations:** 1 Grupo GEPAMOL, Centro de Investigaciones Biomédicas, Universidad del Quindío, Armenia, Colombia; 2 SCIProt, Departamento de Ciencia y Tecnología, Universidad Nacional de Quilmes, CONICET, Roque Sáenz Peña 352, Bernal, Argentina; 3 Grupo GICOC, Universidad del Quindío, Armenia, Colombia

**Keywords:** *Toxoplasma gondii*, Thiazolidinone, Rhoptry proteins

## Abstract

**Background:**

Thiazolidinone derivatives show inhibitory activity (IC_50_) against the *Toxoplasma gondii* parasite, as well as high selectivity with high therapeutic index. To disclose the target proteins of the thiazolidinone core in this parasite, we explored in silico the active sites of different *T. gondii* proteins and estimated the binding-free energy of reported thiazolidinone molecules with inhibitory effect on invasion and replication of the parasite inside host cells. This enabled us to describe some of the most suitable structural characteristics to design a compound derived from the thiazolidinone core.

**Results:**

The best binding affinity was observed in the active site of kinase proteins, we selected the active site of the *T. gondii* ROP18 kinase, because it is an important factor for the virulence and survival of the parasite. We present the possible effect of a derivative of thiazolidinone core in the active site of *T. gondii* ROP18 and described some characteristics of substituent groups that could improve the affinity and specificity of compounds derived from the thiazolidinone core against *T. gondii*.

**Conclusions:**

The results of our study suggest that compounds derived from the thiazolidinone core have a preference for protein kinases of *T. gondii*, being promising compounds for the development of new drugs with potential anti-toxoplasmosis activity. Our findings highlight the importance of use computational studies for the understanding of the action mechanism of compounds with biological activity.

**Electronic supplementary material:**

The online version of this article (10.1186/s12864-018-5223-7) contains supplementary material, which is available to authorized users.

## Background

*Toxoplasma gondii* is a parasite that infects a large variety of mammals -including humans- and represents an important public health concern. *T. gondii* belongs to the *phylum* Apicomplexan and is distributed globally. It is estimated that about one-third of the world’s population is infected. The infection is usually asymptomatic in immunocompetent individuals [1], although some studies report that chronic infection may be associated with changes in behavior and other physiological processes, such as schizophrenia and suicide [2]. Women who acquire the infection during pregnancy transmit the parasite to the fetus by congenital infection, affecting its development, which can be lethal in immunocompromised individuals. In addition, farm animals also get infected and develop cysts in the muscle tissue that are later consumed by humans. Other effects of the infection include malformations in fetuses and spontaneous abortions, especially in ruminants, goats, and sheep [3].

The current treatment for toxoplasmosis presents severe side effects. The generic therapy for the infection is a combination of antifolates. For acute toxoplasmosis infections, the antifolates pyrimethamine (PYR) and trimethoprim are frequently used, together with either sulfadiazine or antibiotics, such as clindamycin. The treatment with PYR causes anemia due to the inhibition of the enzyme dihydrofolate dehydrogenase [4, 5] and, consequently, PYR is usually administered simultaneously with folinic acid (leucovorin) because humans (unlike *T. gondii*) can use exogenous folinic acid for their cells [6]. This treatment is characterized by severe side effects: hypersensitivity, hematological toxicity, teratogenicity, and allergic reactions. Furthermore, the parasite can develop resistance to this treatment and its susceptibility to PYR varies among *T. gondii* strains.

Thiazolidinone derivatives present diverse biological activities [7]. These derivatives present promising pharmacological potential for the treatment of *T. gondii* infections. For instance, Tenório et al. synthesized and elucidated the structure of thiazolidinone derivatives and their in vitro biological activity against *T. gondii* [8]. This group of derivatives had the phenyl, methyl, ethyl and hydrogen groups located at N-3 position thiazolidinones, and nitrobenzene groups substituted the moiety arylhydrazone that is attached to the carbon of the 2-position. Afterwards, to increase the variability of the imine position, De Aquino et al., designed thiazolidinone derivatives with a phenyl substituent at N-3 position showing better values of IC_50_ for both infected cells and intracellular parasites [9]. Also, Carvalho et al. described compounds with the 2-arylhydrazone moiety substituent at *para* position with hydrogen. These compounds showed higher anti-proliferative effect than the previous substituents: chlorine and the nitro group [10]. In addition, Liesen et al. showed that 1,3-thiazolidin-5-yl-acetic acid significantly decreases the percentage of infected cells and the mean number of tachyzoites per cell at concentrations of 0.1, 1, and 10 mM when compared with hydroxyurea and sulfadiazine (standard drugs) [11]. Finally, D’Ascenzio et al. [12] and Carradori et al. [13] explored several different substituents at the N1-hydrazine portion of the thiazolidinone scaffold, ranging from small aliphatic chains to aromatic and bicyclic rings, and the influence of a benzyl group at the lactamic NH of the core upon biological activity.

In this article, we have assessed possible molecular-targets for thiazolidinone derivatives in *T. gondii* and explored the mechanism of action of these compounds through in silico experiments. We chose proteins that play important roles in the survival and virulence of *T. gondii*. The targets chosen were: the small subunit of ribonucleotide reductase (*Tg*RNR2), protein disulfide isomerase (*Tg*PDI), ROP18 kinase protein (*Tg*ROP18), and calcium-dependent protein kinase 1 (*Tg*CDPK1). To evaluate protein-ligand interaction, we performed molecular docking simulations with the ligands reported by D’Ascenzio et al. [12] and Carradori et al. [13]. Finally, we chose a promising target to further characterize its protein-ligand interactions by molecular dynamics (MD) simulations.

## Methods

### Selection and preparation of ligands

**Fig. 1:**
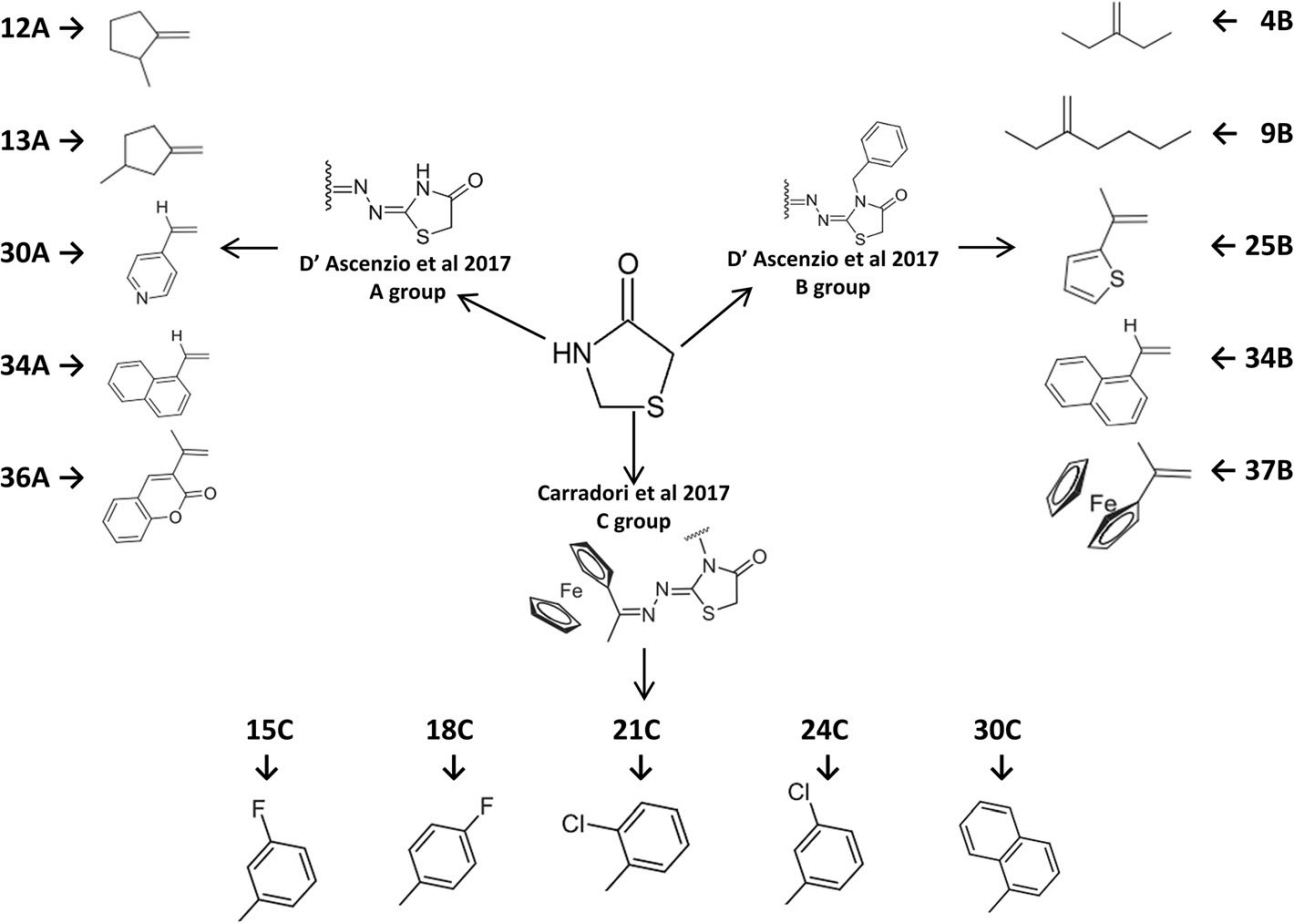
Cores of series A, B and C with their respective substituent groups of the thiazolidinone derivatives tested

The 15 compounds used in this study derive from thiazolidinone. Ten of them were taken from the series A and B presented by D’Ascenzio et al. [12] and other five (serie C) following the compounds proposed by Carradori et al. [13] (Fig. 1). We chose the compounds that reported the highest therapeutic index (TI), defined as TI = median cytotoxic dose (TD_50_)/median inhibitory concentration (IC_50_). As a second criterion, we prioritized molecules with low IC_50_ and low IC_90_ values (Table 1). The structure of the thiazolidinone derivatives was obtained by energy minimization using the Gaussian 09 software [14] at the B3LYP/6-31G* (d,p) basis set and DFT method; the stability of the molecular structures was inspected by checking that the Hessian matrix presented no negative values [15].

**Table 1:** Results of molecular docking simulations for the selected thiazolidinone core derivatives

Derivative	ΔG–*Tg*PDI (kcal/mol)	ΔG–*Tg*RNR2 (kcal/mol)	ΔG–*Tg*ROP18 (kcal/mol)	ΔG–*Tg*CDPK1 (kcal/mol)	TI	IC_50_ (μM)	IC_90_ (μM)
12A	−5.8	−6.1	− 6.1	− 6.6	39	0.9	2
13A	−5.9	−6.3	−6.1	− 6.8	23	24	154
30A	−5.9	− 5.7	−6.0	−6.7	28	20	129
34A	−6.6	−7.7	−8.2	−9.1	144	3.9	31
36A	−8.0	−6.7	− 8.5	− 8.7	70	8	49
4B	−6.6	−6.2	− 6.6	−7.3	16	36	243
9B	−6.2	−5.2	−6.3	−7.5	37	15	121
25B	−7.0	−5.5	−6.9	−8.2	15	37	84
34B	−7.9	−7.5	−8.3	−9.2	15	38	79
37B	−7.8	−6.0	−8.1	−7.8	66	8.5	21
15C	−8.2	−6.3	−8.1	− 8.3	40	8	57
18C	−8.1	−6.0	−8.2	− 8.5	53	6	16
21C	−7.8	−6.2	−8.0	−8.0	36	9	24
24C	−7.9	−5.9	−8.4	−8.2	40	8	17
30C	−8.7	−6.3	−8.9	−10.0	64	5	17

Values of TI, IC_50_ and IC_90_ were taken from D’Ascenzio et al. [12] and Carradori et al. [13]

### Selection and modelling of *T. gondii* proteins

*Tg*PDI was selected because it has a closely related analog that is susceptible to thiazolides. Muller et al. indicated that thiazolides interfere with Protein Disulfide Isomerase of *Neospora caninum* (*Nc*PDI). The interaction was tested by affinity chromatography of *N. caninum* tachyzoite extracts on Nitazoxanide, which is a prototype member of the thiazolidines accepted by the Food and Drug Administration. *N. caninum* is a parasite phylogenetically related with *T. gondii*, and *Nc*PDI is possibly related to the PDI proteins from other microorganisms susceptible to thiazolides [16]. These features make *Tg*PDI an interesting target to consider. The sequence of *Tg*PDI was obtained from ToxoDB [17], code TGME49_211680. The template used for the homology modelling was the human PDI, with PDB code 3F8U (chain A) [18].

*Tg*RNR2 was chosen because it is inhibited by molecules structurally related to the thiazolidinone core. Montazeri et al. described that hydroxyurea and thiosemicarbazones compounds inhibit the small subunit of ribonucleotide reductase (RNR2) of *T. gondii*. Due to this inhibition, the compounds that inhibit *Tg*RNR2 interfere with the DNA synthesis and present anti-*T. gondii* activity. Since these molecules are closely related to thiosemicarbazides and thiazolidinones, it may be possible that molecules containing the thiazolidinone core have similar pharmacodynamics [19], i.e., interacting with the di-nuclear iron center of the RNR2 subunit [20], and therefore we included *Tg*RNR2 for the analysis. The sequence of *Tg*RNR2 was obtained from ToxoDB, code TGGT1_207060. The template used for the homology modelling was the RNR2 of *Plasmodium vivax*, with PDB code 2O1Z (chain A).

It is known that molecules that contain the thiazolidinone scaffold inhibit protein kinases. Literature indicates that thiazolidinones inhibit protein kinases that are relevant for the survival of malignant cells; such as phosphoinositide 3-kinase (PI3K), mitogen activated kinase (MEK), pim kinase1 (Pim-1) and 5′-adenosine monophosphate activated protein kinase (AMPK) [21, 22]. This opens the possibility to find interactions between thiazolidinone derivatives and kinase proteins of *T. gondii*.

The kinase domain of *Tg*ROP18 was selected because this protein is essential for *T. gondii*. Rhoptry proteins, known as ROP proteins, such as *Tg*ROP18, are kinases from the Rhoptry organelles of the parasite that are unique to apicomplexan organisms. They are injected into the host cell in the precise moment of parasite internalization and manipulate the immune response of the host. These roles make *Tg*ROP18 a key factor to modulate the virulence of *T. gondii* [23] and, given that *Tg*ROP18 contains a serine/threonine kinase domain, it has been included in the analysis. *Tg*ROP18 protein is found in the Protein Data Bank with the PDB code 4JRN [24]. However, its crystallization presents missing regions (Gln211 to Ala217, Ala440 to Ile441, Phe453 to Thr456, and Glu549 to Glu554). To complete the structure, we modelled the remaining residues using the crystal structure as a template.

*Tg*CDPK1 was chosen because it is another protein kinase crucial for *T. gondii*. This protein is part of an important kinase-like protein family, involved in essential pathways controlled by calcium. In addition, it is related to the mobility of the parasite and, therefore, to the attachment/invasion to the host cell. These proteins can be found in protozoan parasites, plants, and ciliates, but not in animals or fungi [25]. These serine/threonine kinase proteins have been implicated in specific functions and in the development of distinct stages in the complex life cycles in the apicomplexan parasites [26]. For all the above reasons, *Tg*CDPK1 is an interesting target to explore. The crystal structure of this protein can be found in the Protein Data Bank, with PDB code 3SX9 [27]. However, its structure presents small missing regions (Glu314 to Val318 and Met390 to Gln393). To complete the structure, we modelled the remaining residues using the crystal structure as a template.

The models were made with the SWISS-Model web server [28]. The visual inspection of the built models, the structural alignment between each model and its correspondent template, and the calculation of their root-mean-squared-deviation (RMSD) was performed through UCSF Chimera 1.12 program [29]; Ramachandran plots were generated with the ProFunc web server [30].

### Molecular docking

Ligands and receptor proteins were prepared with the AutoDock Tools v1.5.6 software [31]. The position and size of the search box were set to encompass every residue involved in the catalytic site of each receptor. Molecular docking simulations were performed using the AutoDock Vina v1.1.2 package [32]. We set the exhaustiveness parameter to 20. Then, to validate the results and improve the screening, we performed a re-scoring with the scoring functions DSX-score [33] and X-Score [34]. The poses re-scored were the best ones obtained from Autodock Vina for each thiazolidinone derivative. The complex receptor-ligand interactions (Van der Waals, hydrophobic contacts, and hydrogen bonds) were visualized with the software BIOVIA Discovery Studio Visualizer v16.1.0.15350 [35]. The docked poses were visualized using UCSF Chimera v1.12 [29].

### Molecular dynamics simulations

The result of the previous procedures led us to perform molecular dynamics (MD) simulations of two systems: *Tg*ROP18 bound to ATP (the substrate) and *Tg*ROP18 bound to ligand **30C**. The *Tg*ROP18 complete structure was used as the initial coordinates of the protein. The initial coordinates of **30C** were taken from the docked pose with the best score; the coordinates of ATP and Mg^+ 2^ were generated from the co-crystalized molecules AMP-PNP and Mg^+ 2^ in *Tg*ROP18 crystallization [24]. The systems were solvated in a water box of 97 × 88 × 93 Å^3^. Water molecules were randomly replaced by Na^+^ and Cl^−^ to neutralize the systems and reach the salt concentration of 0.15 M. We used Amber ff14SB force field for the protein and TIP3P model for water molecules. The ATP force-field parameters and charges were obtained from Bryce Group [36]. Force-field parameters of **30C** were generated with MCPB.py [37]. The molecular orbital calculations required for the procedure were performed with Gaussian 09 [14] using the B3LYP/6-31G* basis set.

The systems were minimized with 5000 steepest-descent steps followed by 5000 conjugate-gradient steps. We applied harmonic restrictions to protein and ligand heavy atoms, setting a force constant of 10 kcal/mol/Å^2^. Then, the systems were heated linearly from 0 to 310 K at constant pressure in 250 ps. The temperature was controlled with a Langevin thermostat, with a collision frequency of 1 ps^− 1^. The time-step was set to 1 fs. The pressure was set to 1 bar and was controlled by changing the boundaries of the box isotropically through a Berendsen thermostat, with a pressure relaxation time of 1 ps. We used Particle Mesh Ewald method to calculate long-range electrostatic interactions with an electric cut-off value of 10 Å. Afterwards, for the equilibration stage, five simulations of 200 ps were carried out to remove the restraints gradually. The force constants used for restraining the complex heavy atoms were 5.0, 2.5, 1.0, 0.4, and 0.1 kcal/mol/Å^2^, respectively. Finally, for the production stage, we increased the time-step to 2 fs while constraining bond lengths involving hydrogen atoms with the SHAKE algorithm. In this stage, configurations of the system were saved every 20 ps. For each system, we performed three equivalents MDs of 300 ns with different thermostat random seed. Additionally, we validate two conformations sampled in the MDs (one from MD1 of *Tg*ROP18/**30C** and another from MD3 of *Tg*ROP18/ATP). For this aim, we selected a representative configuration from each MD and repeated the equilibration/production stage. In the end, we have four MDs of each system. All MD simulations were performed with AMBER16 software [38]. The analysis of the trajectories was made with AmberTools18 and self-written software. Figures were created with VMD [39], Gnuplot, and Inkscape software.

## Results

### Structural description of the receptors

We obtained the structure of selected proteins of *T. gondii* to evaluate their inhibition by thiazolidinones. The proteins *Tg*PDI, *Tg*RNR2, *Tg*ROP18, and *Tg*CDPK1 were selected due to their relevance in *T. gondii* and because previous in vitro studies point out they could be inhibited by thiazolidinone derivatives. To obtain the structures of *Tg*PDI and *Tg*RNR2 we used homology modelling; the structures of *Tg*ROP18 and *Tg*CDPK1 were obtained from the Protein Data Bank.

**Fig. 2:**
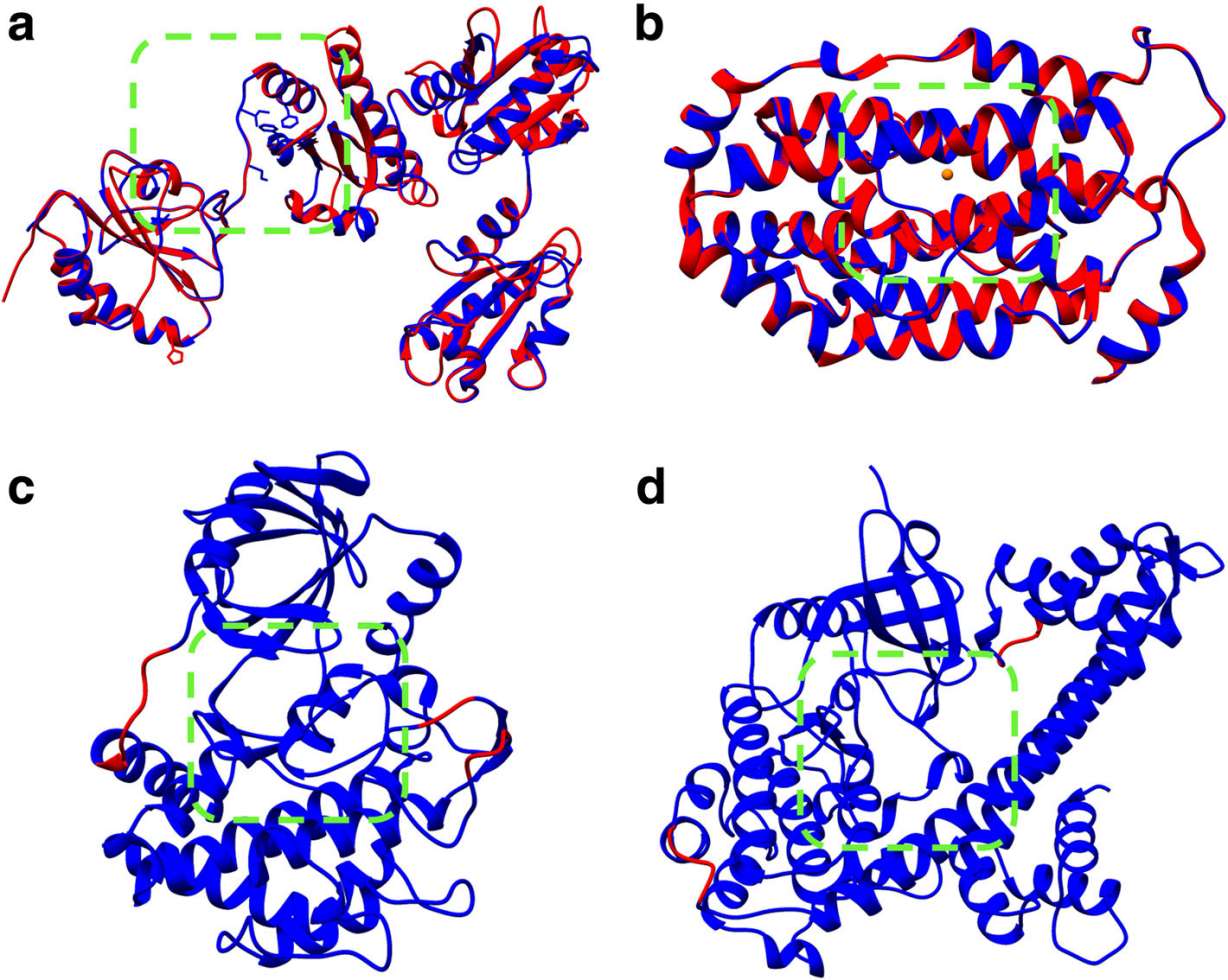
Structural alignments between the models and its templates. **a** PDI model of *T. gondii* (blue) aligned with human PDI (red) with PDB code 3F8U. **b** RNR2 small subunit β model of *T. gondii* (blue) aligned with *Plasmodium vivax* RNR2 subunit β (red) with PDB code 2O1Z. **c** *Tg*ROP18 crystal structure (blue) with PDB code 4JRN; the missing regions modeled are shown in red. **d** *Tg*CDPK1 crystal structure (blue) with PDB code 3SX9; the missing small regions modeled are shown in red. Green squares show active sites evaluated in each target proteins

The *Tg*PDI model presented a structure similarity with the template and conserved physicochemical properties (Fig. 2a). *Tg*PDI sequence had 38% of identity with the template (human PDI) and a coverage score of 0.96. Additional file 1: Figure S1 shows the sequential alignment between *Tg*PDI and its template. The RMSD between the model and the template was 0.27 Å, which indicated that the tertiary structure was preserved. The Ramachandran plot of the model showed that 88% of residues were located in the most-favored regions and 9.9% are placed into the additional regions permitted. QMEAN value is − 3.77. Altogether, results suggest a good quality model (Fig. 2a). The *Tg*PDI model presents semiconserved residues, such as Ile310 and Ser313 (Leu337 and Tyr340 in the template, respectively). These residues were placed at the highly preserved x-linker structure from PDI family, located between b’ and a’ domains. Also, Phe283 and Phe301 (Phe283 and Phe304 in the template, respectively) residues shape a hydrophobic pocket in the a’ domain, where x-linker residues interact with them. Although Val330 (Tyr332 in the template) is not a conserved residue, it does have semiconservative physicochemical properties [40].

The *Tg*RNR2 model conserves at least eight relevant residues in the catalytic site (Fig. 2b). *Tg*RNR2 sequence has 65% of identity with its template (*Plasmodium vivax* RNR2) and coverage of 0.75. Additional file 2: Figure S2 shows the sequential alignment between *Tg*RNR2 and its template. The RMSD between the model and the template was 0.11 Å, which indicates the tertiary structure was not altered (Fig. 2b). The Ramachandran plot showed that more than 90% of the residues were located in the most-favored regions and none in the generously and disallowed regions. The QMEAN value is − 2.08. These data suggest a good quality model. It can be observed from the model that many residues surrounding the di-nuclear iron center of RNR2 are conserved. There residues are: Glu172, His175, Ser176, Glu234, Phe238, Glu268, His271, and Tyr179. The residues are located in the helices αB, αC, αE, and αF.

The structure of *Tg*ROP18 has several kinase residues conserved (Fig. 2c). The catalytic residues from kinases (known as “the catalytic triad”) are present: Lys281, Asp409, and Asp427. We find residues located in the Glycine rich loop (G-loop) that plays an important role to hold ATP (the substrate) in the active site: Gly259, Gly261, Gly262, Phe263, and Val266. There are conserved residues from the A-loop, Phe428 and Gly429 that play a major role in the regulatory mechanism that induces a conformational change that activates the protein for subsequent γ-phosphate transference to the substrate phosphorylation site. Lys411 and Asn414 are located in the catalytic loop. Ala359 is located in the hinge region, which interacts with the adenine ring. Met356, known as gatekeeper, covers a hydrophobic pocket posterior to the ATP location. Finally, Glu300 is a key residue that intervenes in the salt-bridge formation along with Lys281, allowing the breath-like movement of opening and closing the two lobes to let the ATP substrate in and out and enable phospho-transference.

The active site of *Tg*CDPK1 presents kinases catalytic triad: Lys80, Asp174 and Asp195 (Fig. 2d). In addition, the gatekeeper residue of *Tg*CDPK1 is a glycine (Gly128). In general, gatekeeper residues in human kinases have higher volumes; this difference has been exploited for the selective design of drugs against apicomplexan parasites such as *T. gondii* and *N. caninum* [25]. The Ramachandran plot for the *Tg*CDPK1 protein was obtained to have a reference from a crystal structure representation, showing 94.7% of residues located in the most-favored regions.

### Molecular docking

Thiazolidinone derivatives were docked into the protein targets with Autodock Vina (Table 1). The results of the molecular docking suggest that the **30C** derivative presents the best binding affinity in three of the four protein models evaluated (*Tg*ROP18, *Tg*CDPK1, and *Tg*PDI), and it is among the five best derivatives evaluated in the active site of the *Tg*RNR2 model. Moreover, the results of the re-scoring tests performed with DSX-score and X-Score (Additional file 3) present a trend similar to the results delivered by AutoDock Vina, suggesting that **30C** is one of the best docked compounds in the active sites of the evaluated proteins. Therefore, the **30C** molecule was chosen as the thiazolidinone model to evaluate target-ligand interactions.

**Fig. 3:**
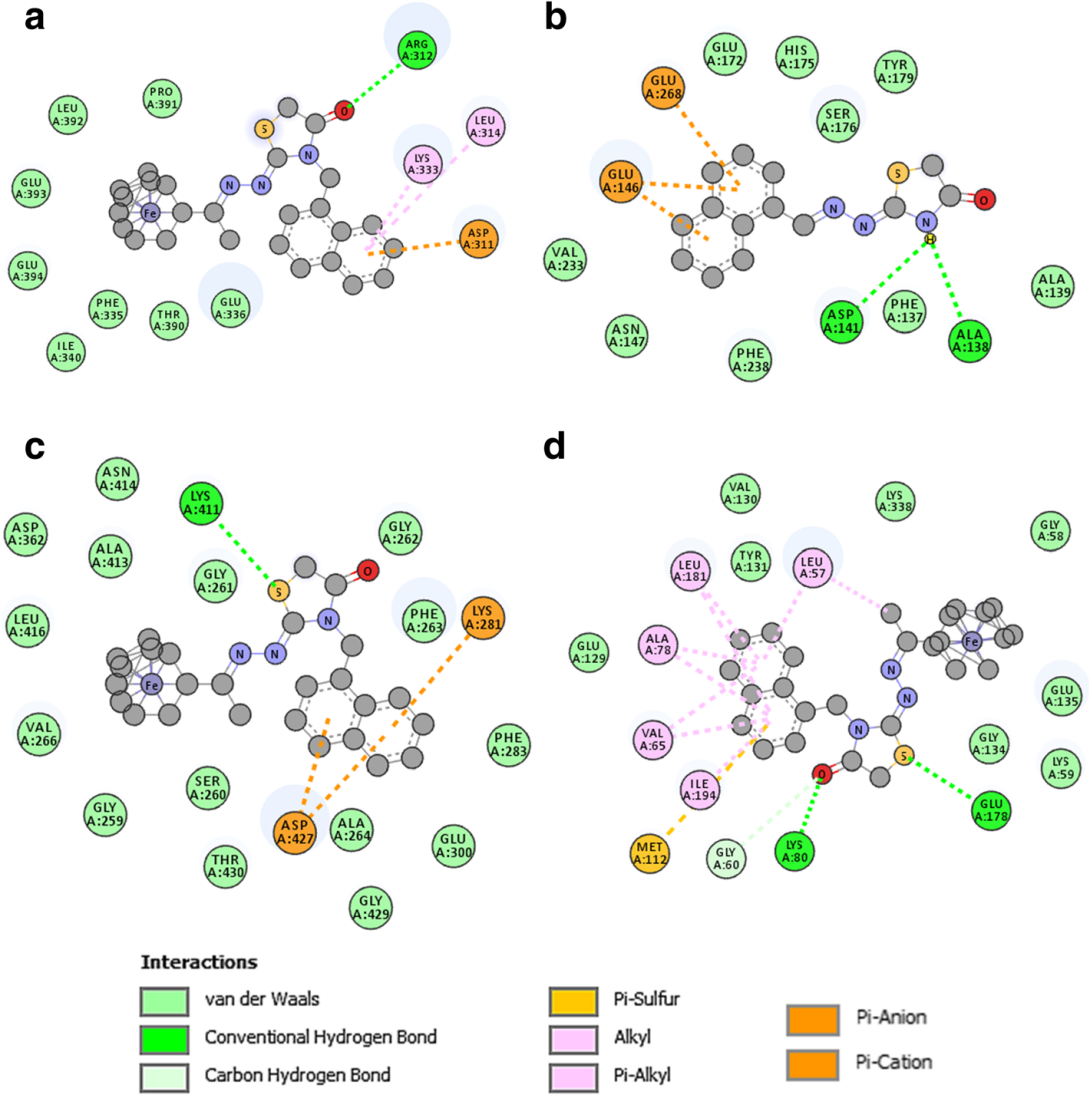
2D interactions of best ligands docked in the binding site of the studied proteins. **a** *Tg*PDI and **30C** ligand complex showing − 8.7 kcal/mol binding-free energy. **b** *Tg*RNR subunit 2 complexed with **34A** presented a value of − 7.7 kcal/mol for the binding-free energy. **c** *Tg*ROP18 complexed with **30C**, showing a binding-free energy of − 8.9 kcal/mol. **d** *Tg*CDPK1 complex with **30C**, showing a binding-free energy of − 10.0 kcal/mol

In the *Tg*PDI model, the **30C** derivative interacts with residues along the x-linker structure and a α-helix from the a’ domain (Fig. 3a). A single H-bond is formed between Arg312 and the oxygen from the carbonyl in the thiazolidinone ring; Asp311, Leu314, and Lys333 make π-ion/π-π interactions with the second N-3 aromatic ring substituted; and residues Phe335, Glu336, Ile340, Thr390, Pro391, Leu392, Glu393, and Glu394 interact by Van der Waals forces with the ferrocene group.

In the *Tg*RNR2 model, the compound with lowest score is **34A**, which shows H-bonds and π-anion interactions for important and conserved residues surrounding the di-nuclear iron center (Fig. 3b). Asp141 and Ala138 stabilize the **34A** through H-bonds aimed at the amine in the thiazolidinone ring; Glu146 and Glu268 show π-anion interactions with the naphthalene moiety of **34A**; Tyr179, Phe137, A139, Asn147, Glu172, His175, Ser176, Val233, and Phe238 interacts through Van der Waals forces with naphthalene moiety and thiazolidinone core of the **34A** derivative. It is interesting to compare the pose of **34A** and **30C**. In the case of the **30C** derivative, the hydrophobic and Van der Waals interactions between the naphthalene moiety and the residues Glu146, Glu172 and Asn147 are conserved; while amino acids such as Asp141, Ala138, Ala139 and Ser176 interact with the ferrocene moiety instead of thiazolidinone core.

In the active site of the *Tg*ROP18, **30C** interacts with residues that stabilize ATP (Fig. 3c). Lys281 and Asp427 present π-ion interactions with the naphthalene substituent; Lys411 forms an H-bond with the sulfur atom in the thiazolidinone core; Gly259, Ser260, Gly261, Gly262, Phe263, Ala264, Val266, Phe283, Glu300, Asp362, Ala413, Asn414, Leu416, Gly429, and Thr430 present Van der Waals interactions with the ferrocene, naphthalene and thiazolidinone moieties.

In the active site of the *Tg*CDPK1, **30C** interacts with the residues Leu57, Val130, Tyr131, Leu181, Ala78, Glu128 and others with its naphthalene moiety, through hydrophobic interactions. Also, it interacts with Gly60, Lys80, and Glu178 through its thiazolidinone core that presents strong interactions as H-bond. Lastly, **30C** interacts with Lys338, Gly58, Glu135, Gly134 and Lys59 with its ferrocene moiety mainly presenting Van der Waals interactions (Fig. 3d).

The general trend of scores suggests that the targets with highest affinity with thiazolidinones are, in decreasing order: *Tg*CDPK1, *Tg*ROP18, *Tg*PDI, and *Tg*RNR2. The target chosen for further evaluation was *Tg*ROP18 due to the low docking scores and its importance in *T. gondii* pathogenesis. We chose the protein *Tg*ROP18 because it is a key virulence factor for *T. gondii* [41, 42]. It controls the intracellular proliferation of the parasite [43, 44], it manipulates the host’s immunity and cell apoptosis [45–47], and it had been studied in our research group in previous works [48–51]. Altogether, the complex selected for further evaluation with molecular dynamics is *Tg*ROP18/**30C**.

### Molecular dynamics simulations

We performed MD simulations of the complex *Tg*ROP18/ATP and *Tg*ROP18/**30C** to characterize their interactions. The system *Tg*ROP18/ATP was used as a reference of the natural biological interaction of the protein. We simulated four replicas of each complex. In the MDs of *Tg*ROP18/ATP; ATP keeps the conformation of AMP-PNP found in the crystal structure. In the MDs of *Tg*ROP18/**30C**; **30C** buries the naphthalene moiety and the thiazolidinone scaffold, improving the contact with the protein and reaching diverse stable structures. One of the final conformations of the *Tg*ROP18/**30C** complex is compact and the substrate has low exposure to the solvent; in the other one *Tg*ROP18 is open and **30C** is more exposed to the solvent.

**Fig. 4:**
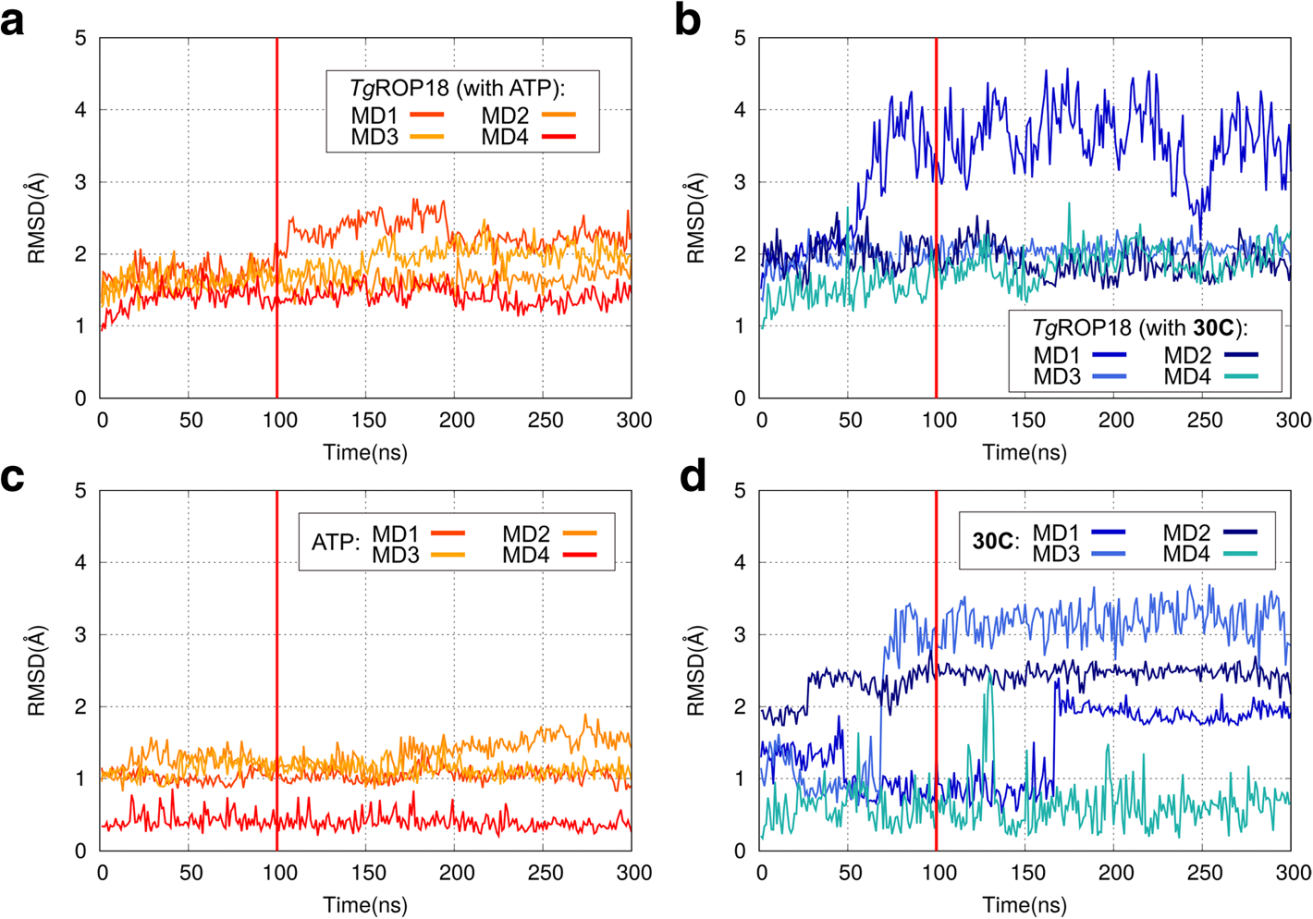
RMSD as a function of the simulation time. In panels **(a)** and **(b)**, plots of the RMSD calculated with the α-carbons of the protein after aligning them to the initial structure with the Kabsch algorithm. In panels **(c)** and **(d)**, plots of the RMSD calculated with all heavy atoms of the corresponding ligand, previously aligning the structures to the initial conformation with the mass weighted Kabsch. Each plot corresponds to one of the four MDs performed with each system. Plots corresponding to the *Tg*ROP18/ATP complex are colored in orange tones, panels **(a)** and **(c)**, and the ones corresponding to *Tg*ROP/**30C** are colored in blue tones, panels **(b)** and **(d)**. The red line at 100 ns indicates the time used for the beginning of the analysis, where the systems are considered equilibrated

In all the MD runs, the systems presented considerable relaxation times before reaching the final structure. For each MD run, we compared snapshots of the trajectory with the initial structure though RMSD in two ways: using the protein backbone (Fig. 4a and b) and using the heavy atoms of the ligand (Fig. 4c and d). All RMSD curves reach a plateau after 100 ns. Therefore, we decided to use the last 200 ns of all MDs in the analyses. It should be mentioned that MD1 of *Tg*ROP18/**30C** complex presents a clear change in the ligand RMSD plot at 167 ns. This jump corresponds to a rotation of a single dihedral angle, which flips the ferrocene group and leaves the interaction unchanged.

### Contact frequency analysis

**Fig. 5:**
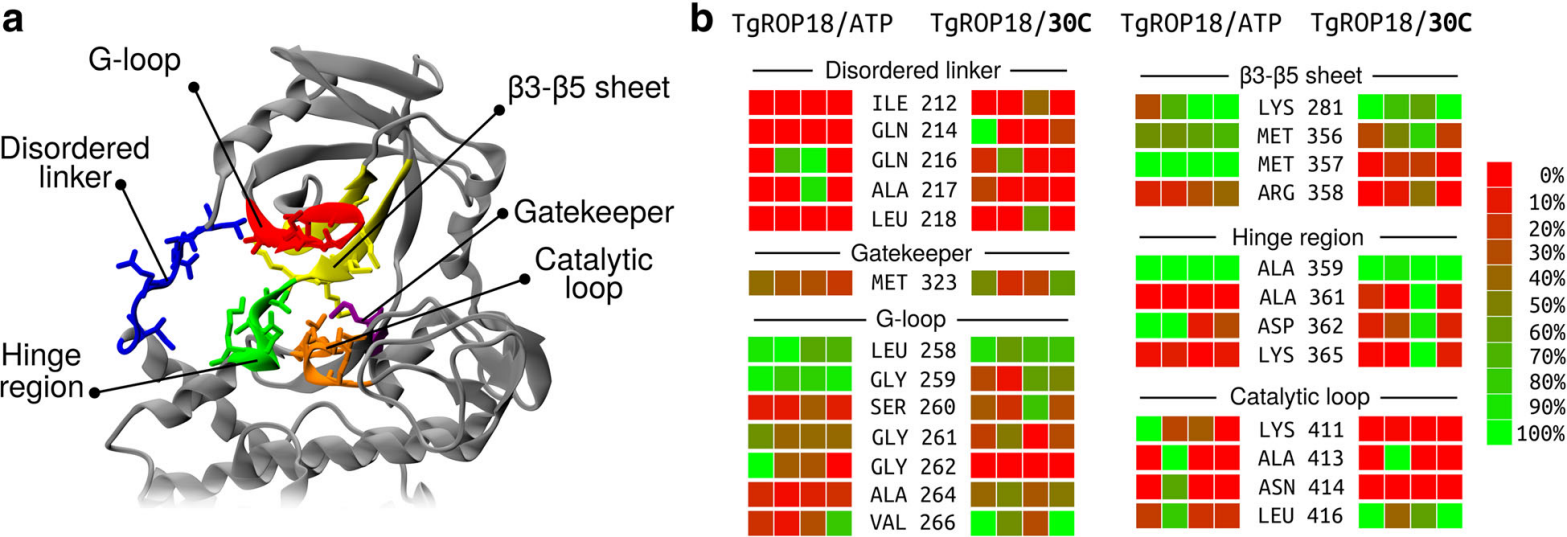
Analysis of contact frequencies in the MD runs of *Tg*ROP18/ATP and *Tg*ROP18/**30C**. In panel **a**, schematic view of the binding site. The sub-structures of the protein are shown in different colors. In panel **b**, contact frequency between the ligand and the residues with stronger contacts. Each square represents the contact frequency of residue in one MD run. The contact frequency of the residues has been colored from red (0%) to green (100%), as shown in the palette. The contacts of *Tg*ROP18/ATP are displayed to the left; the ones of *Tg*ROP18/**30C** to the right. The contacts have been separated by the sub-structure they belong to

We calculated the frequency of contact of each residue with the ligand, ATP or **30C**. It was considered that a contact exists when an atom of a residue was closer than 2.5 Å to any atom of the ligand. In the interest of clarity, residues were grouped according to their spatial localization: disordered linker (residues 211–218), G-loop (residues 258–266), β3-β5 sheet (residues 276–284 and 353–358), hinge region (residues 359–365), gatekeeper residue (Met323), and catalytic loop (residues 411–416). This classification follows the specifications of the crystal structure and is depicted in Fig. 5a.

The most important contacts with ATP and **30C** are shown in Fig. 5b, displayed by group and separated by MD run. The residues that contact ATP interact with different moieties of ATP. The residues that contact the adenine portion are: Leu258 and Val266 (G-loop); Met356 and Met357 (β3-β5 sheet); Ala359 (hinge region); and Leu416 (catalytic loop). In addition, residues that interact with the ribose moiety are: Gly259 (G-loop); Asp362 (hinge region); and in some MDs Ala413 (catalytic loop), Gln216 and Ala217 (disordered linker). Lastly, the triphosphate portion interacts with: Gly261 and Gly262 (disordered linker); Lys281 (β3-β5 sheet); and less frequently with Asn414 and Lys411 (catalytic loop).

On the other hand, with respect to **30C** (Fig. 5b), the residues that contact thiazolidinone portion are: Ser260, Gly261, and Ala264 (G-loop); and Lys281 (β3-β5 sheet), that interacts with the carbonyl group of thiazolidinone scaffold and carboxyl group of Glu300. In addition, residues that interact with naphthalene moiety are: Val266 (disordered linker); Met356 (β3-β5 sheet); Met323 (gatekeeper); Ala359 (hinge region); and Leu416 (catalytic loop). Lastly, the ferrocene portion interacts with: Leu258 and Gly259 (G-loop); Ala361 (hinge region); and four residues Gln214, Gln216 (disordered linker) and Asp362, Lys365 (hinge region) that eventually get close and form H-bonds with each other. As can be noted, these interactions differ from the ones obtained with molecular docking. This is because the moieties have changed their location from the initial pose, maximizing their interactions and minimizing the solvent exposure of hydrophobic moieties.

We observe that, in general, the contacts of ATP or **30C** do not change along the MD runs. To this aim, we divided each simulation into four parts of 50 ns repeated the contact analysis independently in each 50 ns part. In Additional file 4: Figure S3, we show residues that had more than 50% of contact in at least one of the 50 ns portions of the MDs. In general, the contact frequencies are preserved and show no trend with the simulation time. Nevertheless, there are some exceptions that should be mentioned. In the system *Tg*ROP18/ATP, there are changes in the residues: Gln216 and Ala217 (disordered linker); Gly262 (G-loop); Lys281 (β3-β5 sheet); Met323 (gatekeeper); and Lys411 and Asn414 (catalytic loop). In the system *Tg*ROP18/**30C**, there are changes in: Gln214 (disordered linker) and Ala361 (hinge region). In addition, some contacts present variance along the MDs without a trend; thus, indicating more labile interactions.

Contact frequencies can vary between MD runs of the same complex. Some contacts are strong and exist with high frequency in three or four MDs of a complex; other contacts are weaker and present high frequency in only one or two MDs. In the case of *Tg*ROP18/ATP, the strong contacts are Leu258, Gly259, Met356, Met357, and Ala359; while moderate contacts are Gln216, Ala217, Gly261, Gly262, Val266, Asp362, Lys411, Ala413, Asn414, and Leu416. In the case of *Tg*ROP18/**30C**, the strong contacts are Leu258, Val266, Lys281, Ala359, and Leu416; while moderate contacts are Gln214, Gln216, Leu218, Gly259, Ser260, Gly261, Ala264, Met323, Met356, Ala361, Asp362, Lys365, and Ala413.

Some contacts are unique to a certain ligand; others are common to both. The residues that exclusively contact with ATP are: Ala217 (disordered linker); Gly262 (G-loop); and Lys411 and Asn414 (catalytic loop). The residues that contact with **30C** are: Gln214 and Leu218 (disordered linker); Ala264 (G-loop); and Ala361 and Lys365 (hinge region). On the other hand, the residues that present contact with both residues are: Gln216 (disordered linker); Leu258, Gly259 and Val266 (G-loop); Lys281 and Met356 (β3-β5 sheet); Met323 (gatekeeper); Ala359 and Asp362 (hinge region); and Ala413 and Leu416 (catalytic loop). These contacts indicate that the overall binding site is conserved but presents some differences. In general, **30C** is more buried allowing stronger interactions the G-loop (Ala264 and Val266) and the disordered linker (Leu218). In addition, this enables the interaction between Gln214 and Lys365 that surround **30C** ferrocene moiety when they bind. Conversely, **30C** can’t form interactions with polar residues of the catalytic loop (Lys411 and Asn414).

### Molecular mechanics-generalized born surface area

Results suggest **30C** could bind to *Tg*ROP18 acting as a competitive inhibitor of ATP. The binding-free energy of the ligands was estimated through molecular mechanics-generalized Born surface area (MM-GBSA), using the single MD approach without entropy calculation. We obtained an average binding energy of − 11.5 ± 10.3 kcal/mol for ATP and − 29.0 ± 4.4 kcal/mol for **30C**. The value obtained for **30C** suggests it can bind *Tg*ROP18. This value was compared with the ones obtained by Lyne et al. [52]. They used MM-GBSA to evaluate the binding of ligands to four kinases, which rendered linear fits of the predicted-experimental data. From the reported curves, we calculated the pIC_50_ value that corresponds to − 29.0 kcal/mol (complex *Tg*ROP18/**30C**). The obtained values range from 4.5 to 8.1. These values, even if they present large variance, are in agreement with the ones reported by Carradori et al., who studied the ability of the **30C** ligand to inhibit in vitro tachyzoites of *T. gondii* and reported a pIC_50_ of 5.3 (IC_50_ of 5 μM) [13]. On the other hand, the comparison of MM-GBSA values obtained herein suggests that **30C** could be a competitive inhibitor of ATP. However, even if the results support this idea, they are not conclusive due to two reasons. First, because MM-GBSA method is sensitive to the ligand charge model [53] and we employed different charge models for ATP and **30C** (RHF/6–31 + G* and B3LYP/6-31G* sets, respectively). Second, because the precision of the method makes the difference in values not very significant [54].

**Fig. 6:**
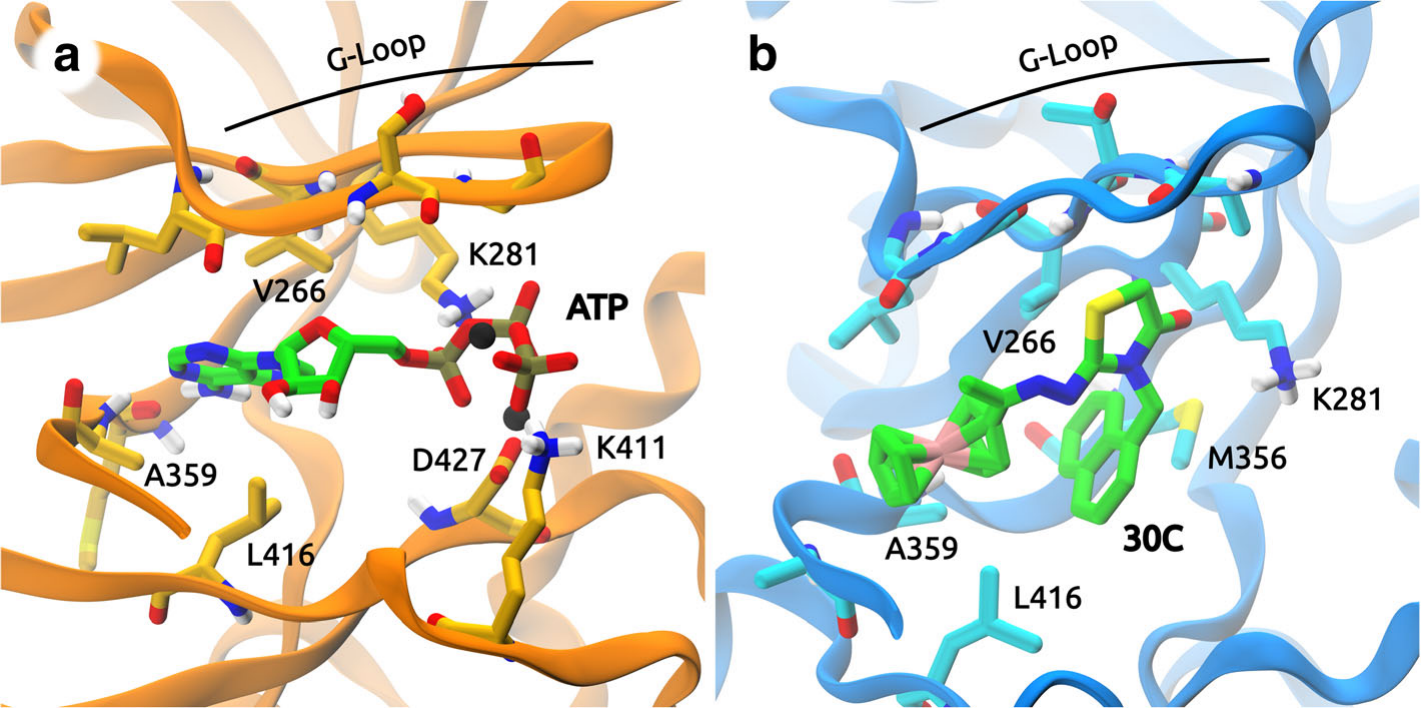
Residues that contribute to the binding-free energy of the ligands. In panel **a**, *Tg*ROP18/ATP. Mg^+ 2^ ions are shown as black spheres. In panel **b**, *Tg*ROP18/ATP. In both panels, only polar hydrogen atoms are shown to highlight their interactions

Residues that contribute to the binding free energy were obtained with free-energy decomposition per residue [55]. Figure 6 shows schematic structures of the binding sites of ATP and **30C**. The most important residues for ATP are: Leu258, Ser260, Gly261, and Val266 (G-loop); Lys281 and Met357 (β3-β5 sheet); Ala359 (hinge region); Lys411 and Leu416 (catalytic loop); and Asp427. The contributions in ATP binding are mainly due to electrostatic interactions. The most relevant residues for **30C** ligand are: Leu258, Gly259, Ala264, Arg265 and Val266 (G-loop); Met356 (β3-β5 sheet); Ala359 and Ala361 (hinge region); and Leu416 (catalytic loop). Their contribution is mainly due to Van der Waals interactions and non-polar solvation. The residue Lys281 (β3-β5 sheet) hinders the binding of **30C**, due to polar solvation energy. As expected, the residues that contribute to the binding free energy had also been found by the contact analysis. However, the residue Asp427, important for ATP stabilization through the indirect interaction with Mg^+ 2^, was not found in the former analysis due to the distance threshold.

### Principal components analysis

**Fig. 7:**
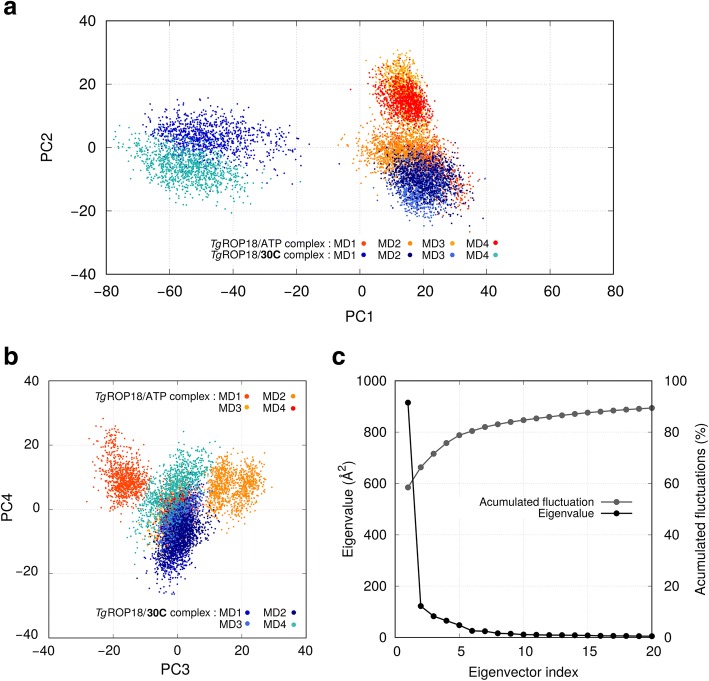
Projections of all MD runs onto the PC-modes of the concatenated PCA. In panel **a**, projection onto the 1st and 2nd PC-mode. In panel **b**, projection over the 3rd and 4th PC-mode. Dots corresponding to the complex with ATP are colored in orange tones while those corresponding to **30C** are colored in blue tones. In panel **c**, plot of the eigenvalues and fluctuation accumulation vs the PC-mode index. Only 20 PC-modes are displayed from the 1053 obtained

To characterize the structural differences between *Tg*ROP18/ATP and *Tg*ROP18/**30C**, we performed the principal component analysis (PCA) method. All the eight MD runs were concatenated and aligned [56]. The covariance matrix was calculated from the positions of the α-carbons of the enzyme, skipping the first and last five residues to avoid noise of ending regions. This left 351 atoms in the analysis and a coordinate space of dimension 1053. The analysis of the accumulated square fluctuations in the PC-modes indicated that these modes contain 75.7% of the total fluctuations of the system (Fig. 7c). In addition, the eigenvalues of the modes decay rapidly. These data indicate that four eigenvectors are enough to explain the variance of the samples.

The trajectories were projected onto the first four PC modes (Fig. 7a and b). The MDs of *Tg*ROP18/ATP sample three clusters of points, indicating three states: MD1 samples around {20, − 5, − 15, 10}; MD2 around {13, − 3, 18, 8}; and MD3/MD4 sample around {15, 15, 0, 0}. These states present high overlap in the 1st and 2nd mode and sample more diversely in the 3rd and 4th mode. In general, each MD run populates a cluster of points and no simulations jump between clusters, except for MD2 that samples two small clusters that we classified into only one state (Fig. 7b). This observation suggests that each MD run samples around single conformation of *Tg*ROP18 (or two in the case of MD2). In addition, MD4, which was started from a representative structure of MD3, samples around the same conformation as MD3 and therefore validates the sampling of that state.

On the other hand, the MDs of *Tg*ROP18/**30C** sample two states: MD1/MD4 sample around {− 50, 0, 0, 0}; and MD2/MD3 sample around {20, − 10, 0, − 5}. These states, contrary to *Tg*ROP18/ATP, present high overlap in the 3rd and 4th mode and samples more diversely in the 1st and 2nd mode. As in the case of TgROP18/ATP, each MD samples around a conformation of the protein. In this system, MD4 was initiated with a representative structure of MD1. We note that the regions sampled by MD1 and MD4 have overlap and resemble to some extent, but they can be unequivocally distinguished in the 2nd and 4th PC-mode. The inspection of the structures of both MDs reveals these differences are due to the position of the disordered linker, which is intrinsically highly mobile, and the β4a/β4b sheet [24], which is far from the binding pocket.

Each PC-mode can be described by concerted changes in the sub-structures of *Tg*ROP18. To describe the motions related to the PC-modes, we have generated coordinates of structures that sample along the PC-modes (Additional file 5: Figure S4). The *Tg*ROP18 structure presents two domains termed N-lobe (residues 209–360) and C-lobe (residues 186–208 and 361–548). The 1st PC-mode represents a hinge motion between the N-lobe and the C-lobe, where domains are near rigid bodies (Additional file 5: Figure S4a). The 2nd PC-mode represents three concerted changes: a lid-like motion of the β4a/β4b sheet (residues 337–342 and 345–350), the rotation of the C-helix (residues 283–310), and the displacement of the disordered linker (Additional file 5: Figure S4b). The 3rd PC-mode also represents concerted changes: a lid-like motion of the C-helix, a rearrangement of the disordered linker, and the opening of the loop formed by residues Val437-Arg451 (Additional file 5: Figure S4c). Finally, 4th PC-mode represents a pseudo-rotation that involves the G-loop, the β4a/β4b sheet, the C-helix, and the disordered linker (Additional file 5: Figure S4d).

The structural representation of the PC-modes allows an intuitive characterization of states of *Tg*ROP18/ATP. In this sense, the sampling of *Tg*ROP18/ATP along the 2nd, 3rd and 4th modes can be thought as natural motions of the Michaelis complex. These movements involve the displacement of the disordered linker, which is in agreement with the poor definition of electron density in that region of the crystal structure. In addition, the lack of sampling along the 1st PC-mode indicates that that in the MDs of *Tg*ROP18/ATP the enzyme does not undergo a wide inter-lobe hinge motion.

### Dynamic domains analysis

PCA revealed *Tg*ROP18/**30C** has two states: one that overlaps with the conformations sampled by *Tg*ROP18/ATP and another clearly different. In addition, we observed that the PC modes involved rigid-body like rearrangements of the substructures in *Tg*ROP18. Therefore, we aimed to identify dynamic domains that could explain the difference between the conformations sampled by *Tg*ROP18/**30C** and those sampled by *Tg*ROP18/ATP. To this aim, we obtained 50 structures of MD1/MD4 of *Tg*ROP18/**30C**, 50 from MD2/MD3 of *Tg*ROP18/**30C,** and 50 structures from all the MDs of *Tg*ROP18/ATP. These groups of structures were compared with the DynDom software [57], that models the difference between conformations as screw motions.

**Fig. 8:**
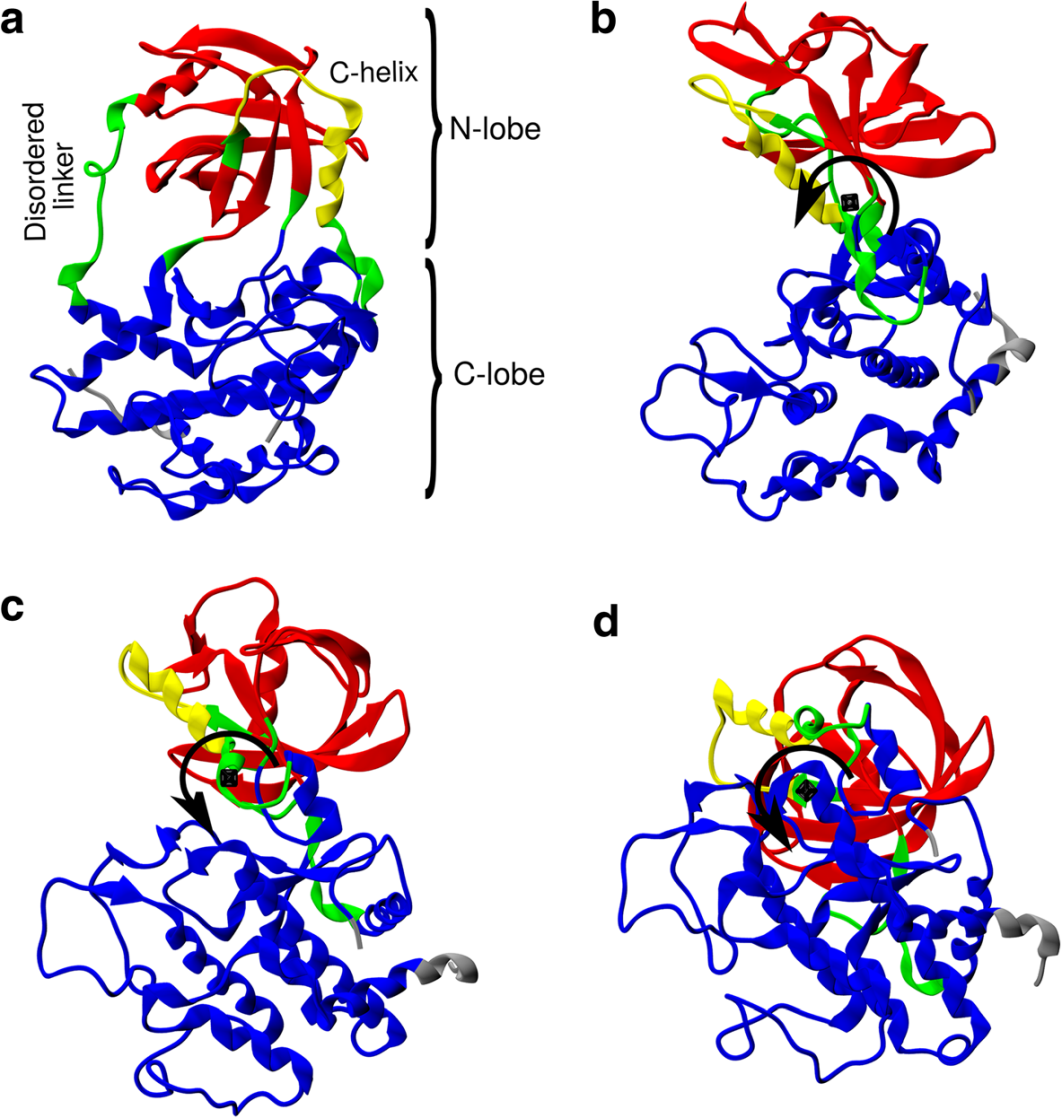
Dynamic domain analysis to compare *Tg*ROP18/ATP with MD1/MD4 of *Tg*ROP18/**30C**. In panel **(a)**, domains identified by DynDom server (blue, yellow and red) and bending regions (green). In panels **(b)**, **(c)**, and **(d)**, views parallel to the screw axes that model the difference between conformations. Black rotation arrows indicate the calculated screw movement

The comparison of the state sampled by MD1/MD4 of *Tg*ROP18/**30C** with the one sampled by *Tg*ROP18/ATP predicted three domains (Fig. 8a): the C-lobe (residues 189–215, 310–326, and 360–539), the C-helix subdomain of the N-lobe (residues 283–302), and the remaining residues of the N-lobe (residues 223–282 and 327–359). Upon the screw motion, the domains presented an RMSD of 1.6 Å, 1.4 Å, and 1.4 Å for the C-lobe, C-helix, and N-lobe, respectively. The low RMSD values indicate the modelling is successful. The screw motion between the C-lobe and the N-lobe (Fig. 8b) presents a closure of 95% and requires a rotation of 34°. The residues identified to bend are from the disordered linker (residues 209–223), the hinge region (residues 359–360), and residues 326–327. On the other hand, the screw motion between the C-lobe and the C-helix (Fig. 8c) has a closure of 97%, requires a rotation of 58° and a translation of 0.9 Å. The residues that bend in this motion are from the C-helix (residues 302–311). Lastly, the motion between the N-lobe and the C-helix (Fig. 8d) has a closure of 87% and requires a rotation of 40° and a translation of 2.4 Å. In this case, the residues that bend are from the β3-β5 sheet (residues 281–282).

The comparison of the state sampled by MD1/MD4 of *Tg*ROP18/**30C** with the one sampled by MD2/MD3 of *Tg*ROP18/**30C** renders results comparable to the ones shown above. Two domains are found: one corresponding to the C-lobe (residues 190–211, 308–325, and 360–539), and one corresponding to the entire N-lobe with the C-helix included (residues 222–307 and 326–359). In this case, the domains presented an RMSD of 1.6 Å and 2.5 Å for the C-lobe and N-lobe, respectively. The screw motion between the domains has a closure of 95%, requires a rotation of 34°, and a translation of 1.3 Å. The residues that bend are from the disordered linker (residues 211–222), the hinge region (residues 359–360), the C-helix (residues 307–310), and residues 325–326. We find that the domain that corresponds to the C-lobe is analog to the one found in the previous DynDom analysis. In addition, the high RMSD value of the N-lobe domain indicates the model is not fitting that well. Also, the N-lobe domain is analog to the merging of C-helix and N-lobe domain found previously. That being said, the data indicates that the domains identified in the two comparisons are approximately equivalent.

In addition, we checked if domains could be defined with several other combinations of MDs. The comparison of the MDs of *Tg*ROP18/ATP with each other does not render domains. The comparison of the state sampled by MD2/MD3 of *Tg*ROP18/**30C** and the one sampled by *Tg*ROP18/ATP also does not render domains. This indicates that, in these cases, the intradomain and interdomain displacements are comparable and, thus, no domains can be identified.

Finally, the PC-modes can be related to the DynDom analysis. The 1st PC-mode, which accumulates 58.4% of the sampled fluctuation, represents the C-lobe/N-lobe hinge movement found in both DynDom analyses. The 2nd, 3rd, and 4th PC-modes, that include an additional 17.3% of the fluctuations, include movements of the disordered linker, the C-helix, and the β4a/β4b sheet. This is also in agreement with the DynDom analysis. First, because C-helix was recognized as a domain; and second, because the disordered linker was identified as a bending region, which is consistent with the diverse displacements it can undergo.

### Backbone correlation analysis

**Fig. 9:**
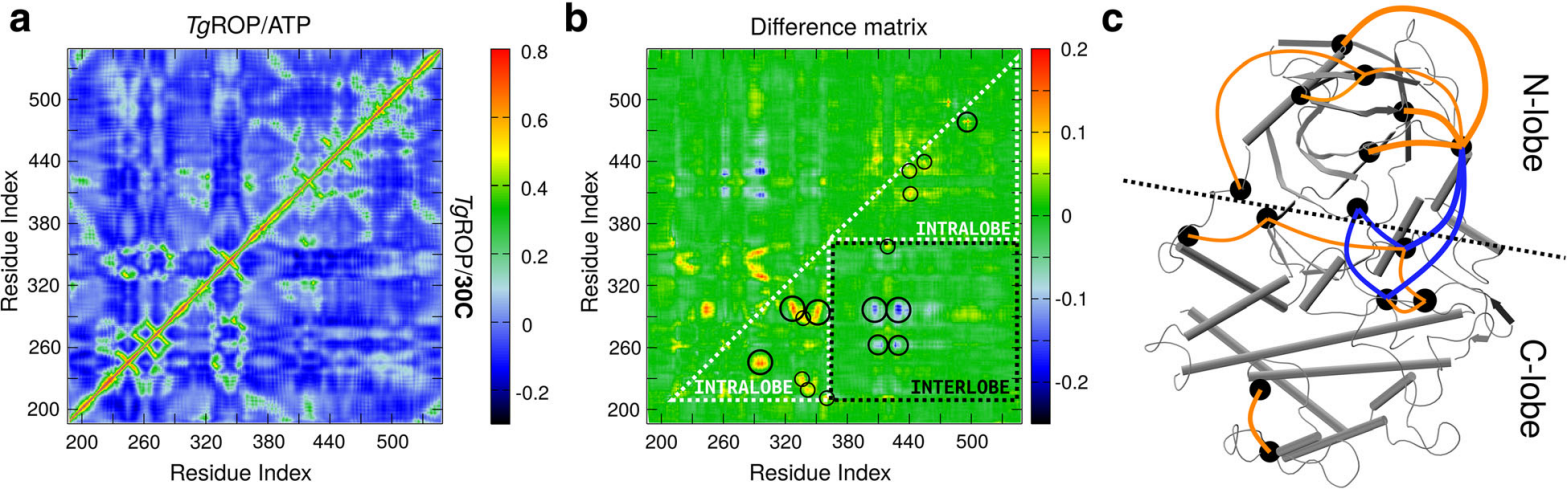
Analysis of correlation between α-carbons of *Tg*ROP18. In panel **a**, heatmaps of the average correlation between α-carbons of *Tg*ROP18/ATP (upper triangle) and *Tg*ROP18/**30C** (lower triangle). The average was obtained with the four MD trajectories. In panel **b**, the difference between the maps of panel *Tg*ROP18/ATP and *Tg*ROP18/**30C**, i.e., **30C** map minus ATP map. We exploited the symmetry of the matrix to show extra information. The upper triangular matrix was left raw. In the identical lower triangular matrix, spots that indicate change of correlation are marked with circles. White triangles and black square marks the region corresponding to intra-lobe and inter-lobe correlation, respectively. In panel **c**, 3D graph of the change of correlation of *Tg*ROP18/**30C** that was displayed in panel **(b)**. Regions of the protein are displayed as nodes; correlation changes are marked as connections indicating increase (orange) or decrease (blue) of correlation

To further characterize the systems, we performed correlation analysis between α-carbons atoms. For each system and each MD run, we obtained the displacement vectors between frames of the α-carbons. Then, the correlation per residue-pair was computed as the average of the dot product of the displacement vectors. Correlations matrices are displayed as heat maps in Fig. 9a. The correlation maps of *Tg*ROP18/ATP and *Tg*ROP18/**30C** are very similar. The correlation values are found between − 0.3 and 0.8. The presence of inverse diagonals with high correlation reflects the multiple antiparallel β-sheets that the structure of *Tg*ROP18 contains; on the other hand, the increase of correlation nears the diagonal of the matrix marks α-helices in *Tg*ROP18.

To assess the effect of **30C**, we calculated the difference between the correlation matrices of *Tg*ROP18/ATP and *Tg*ROP18/**30C** (Fig. 9b). The difference matrix shows few non-zero spots that amount to values from − 0.20 to 0.25 (circles in Fig. 9b). This implies there are no major differences in the correlations of single residue pairs.

On a different approach, the difference matrix was divided into two regions (Fig. 9b): the ones that correspond to residues from the same lobe (i.e., regions with intra-lobe correlations) and the ones that correspond to residues from different lobes (i.e., regions with inter-lobe correlations). The two white triangles represent the intra-lobe correlations of N-lobe (residues 209–360) and a part of the C-lobe (361–548); the black square represents inter-lobe correlations. Interestingly, the spots in the matrix show intra-lobe correlations increases while inter-lobe correlation decrease. In the interest of clarity, Fig. 9c displays these changes of correlation in the structure of *Tg*ROP18 indicating when the pair-wise correlation increases (orange) or decreases (blue).

This analysis shows some regions are more affected by the change of correlation. These regions can be visually appreciated in Fig. 9c as the nodes with many connections. The regions that are affected the most are, in decreasing order: the C-helix, the activation loop, the catalytic loop, and the β4a/β4b sheet. On the other hand, it is interesting that some changes of correlation are found in residues that are far from the binding site, such as the correlation between residue Cys478 and residues Leu492-Pro498, from the C-lobe. A careful inspection of the correlation matrices of MD1/MD4 and MD2/MD3 *Tg*ROP18/**30C** (data not shown) indicates that the changes in correlations are mainly due to the state samples in MD1/MD4. The conformation sampled in MD2/MD3 only corresponds to the decrease in correlation of the G-loop (small blue spots in Fig. 9b) while the conformation sampled in MD1/MD4 corresponds to the rest of the changes of correlation (large blue spots and orange spots, in Fig. 9b). These are consistent with previous analyses. PCA and DynDom analyses showed that **30C** induces a more open conformation in MD1/MD4 of *Tg*ROP18, while it does not change the conformation of *Tg*ROP18 in MD2/MD3.

## Discussion

### Preparation of models of possible drug-targets of thiazolidinones in *T. gondii*

An inherently difficult aspect of drug development is identifying drug-targets and elucidating the mode of action of a given compound. To identify targets, biochemical methods (e.g., pull-down studies with immobilized drugs) and genetic approaches (e.g., analysis of resistant parasite strains) are employed [58]. An ideal drug-target is expressed solely within the pathogen or has a low degree of similarity to homologous host proteins and carries out a functional activity that is essential for the parasite’s survival. Computational tools are valuable in identifying suitable targets. They include functional, structural, and in vitro studies and provide valuable information on the interaction of selected targets with ligands, in general functional inhibitors. Moreover, the quality of the models of the targets identified is also important to obtain results close to the actual protein receptor targets. The Swiss-Model server uses a homology modelling method with quality evaluation being the last step. The QMEAN scoring function is the criteria of reference used to estimate the global quality of the query models; this score reflects the predicted global model reliability [59]. Ramachandran plots map the wealth of conformations of the polypeptide backbone (displaying the values of the φ and Ψ angles) and are widely used to see whether the main chain torsion angles are stereochemically feasible in a protein model. Thus, it predicts possible clashes in their structure and provides a simple view of their conformation of the φ - ψ angles within distinct regions in the Ramachandran plot, where each region corresponds to a particular secondary structure [60]. The results of analysis of Ramachandran plots and the QMEAN score suggest that, in general, the two predicted models chosen (*Tg*PDI and *Tg*RNR2) presented a quality acceptable for their use in studies of molecular docking and molecular dynamics.

### Insight of the effect the 30C derivative in the active site of the *Tg*ROP18 protein

Several studies suggest that compounds with thiazolidinone core have a potent biological activity, such as anti-inflammatory, antibacterial, anticancer, antiviral, antiparasitic, among others [7, 22, 61]. Thiazolidinone could be considered an in vitro hit against *T. gondii* [62]. The study of its pharmacodynamics is important to enhance the chances to achieving a lead molecule that succeeds in animal models.

Molecular docking is a computational simulation approach where a candidate ligand is bound to a receptor. The method predicts the preferred orientation of binding the ligand to form a stable complex. Molecular docking is used to predict the affinity and activity of binding of the small molecule to their protein targets by using scoring functions. Hence, docking plays an important role in the rational design of drugs. The sensitivity of docking calculations shows that even small changes in the ligand conformation can lead to large differences in the scores of the resulting docked poses [63, 64].

We explored the active sites of four different proteins of *T. gondii* that could be involved with thiazolidinone core interaction. The results of re-scoring performed with DSX-score and X-Score, reveal a tendency of the **30C** derivative to be present among the best five compounds for each protein (Supplementary material). For this reason values delivered by Autodock Vina for the binding-free energies are accepted.

Table 2 shows more clearly, that **30C** was the derivative that presented the best values of binding-free energy (values highlighted in bold). This results suggest that the **30C** derivative would be presenting a multi-target effect since it is among the best binding-free energies presented by the derivatives tested in the active sites of the proteins evaluated, exhibiting great affinity for kinase domain proteins, like *Tg*ROP18 and *Tg*CDPK1.

**Table 2:** Summary of the best docked compounds in the ligand binding sites of the studied proteins of *Toxoplasma gondii*

ΔG–*Tg*PDI (kcal/mol)	ΔG–*Tg*RNR2 (kcal/mol)	ΔG–*Tg*ROP18 (kcal/mol)	ΔG–*Tg*CDPK1 (kcal/mol)
**30C (− 8.7)**	34A (− 7.7)	**30C (−8.9)**	**30C (−10.0)**
15C (−8.2)	34B (− 7.5)	36A (− 8.5)	34B (−9.2)
18C (− 8.1)	36A (− 6.7)	24C (− 8.4)	34A (− 9.1)
36A (− 8.0)	**30C (− 6.3)**	34B (−8.3)	36A (− 8.7)
24C (− 7.9)	15C(− 6.3)	34A (− 8.2)	18C (− 8.5)

To select a drug-target that meets the conditions mentioned, few research studies motivate the exploration of possible inhibitors of *Tg*ROP18 [65]. However, recent studies report that *Tg*ROP18 has interactions with human proteins involved in the immune response against *T. gondii*. Furthermore, it is a key factor for the control of virulence and the development of toxoplasmosis [43, 66]. We study the interactions in the *Tg*ROP18/**30C** complex by molecular MD simulations, although *Tg*CDPK1 is also a protein to consider for future studies.

We found that **30C** could induce two conformations in *Tg*ROP18. It is possible that the two conformations are part of the binding procedure of **30C**, i.e., the open conformation would represent a first step in binding that could lead a close conformation; the closed conformation found here is similar to that resulting from the binding of ATP, in which flexibility is induced in the two lobes, causing their closure and consequently a rearrangement of their C-helix. Therefore it can be suggested that the second conformation of **30C** within *Tg*ROP18 would be imitating the effect of the natural substrate [67]. In 300 ns of MD4 we have not detected large changes in the structure reached by MD1/MD4 or MD2/MD3, but simulation times required for the change in conformation may be much longer. In this sense, the reason why we found the two conformations might be because the bias of the initial structure allowed the system to reach different free-energy minima. More specialized simulations would be needed to test this hypothesis. For example, steered molecular dynamics and umbrella sampling simulations have been effective to describe the release of ligands from their receptors [68]. Further, these methods could be used to pull out 30C from both states and assert if they are two different binding modes or two stages in a single binding procedure.

**Fig. 10:**
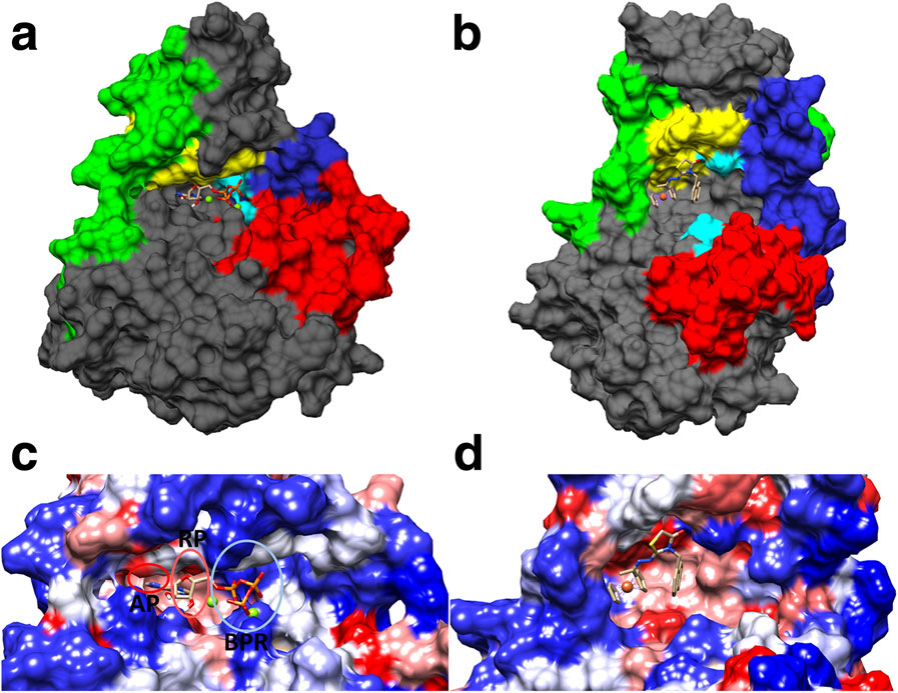
Structural changes on the *Tg*ROP18 protein caused by the action of the **30C** ligand in one of the two possible states. Representative structures of the ATP-*Tg*ROP18 **(a)** and *Tg*ROP18/**30C (b)** complexes after the MD simulations; the G-loop is show in yellow, the disordered region is show in green, the residuals of the catalytic triad are shown in cyan blue, the A-loop is show in red and the Chelix is show in blue. The regions in gray are schematics. **c** Active site of the *Tg*ROP18 model protein, delimited by adenine pocket (AP), ribose pocket (RP), and binding phosphates region (BPR). From left to right, the active site is described with the hydrophobic AP that interacts with hydrophobic substituent groups, and RP that might interact with electron donor groups, and describes the BPR with a hydrophilic profile and tends to form H-bonds with acceptor atoms. **d** Change over the electrostatic surface of the active site of *Tg*ROP18 caused by the **30C** ligand after the MD simulation

Some residues from the *Tg*ROP18 active site interact with **30C** to form and stabilize the receptor/ligand complex. These residues coincide with the ones that stabilize ATP. We found that the stabilization of **30C** in the *Tg*ROP18 active site is principally given by non-polar solvation and Van der Waals interactions. Within the active site of protein kinases some common regions are described where the natural ATP substrate fits in, with one pocket for each of the characteristic groups that build it, such as Adenine Pocket and Ribose Pocket, which have hydrophobic surface, and Binding Phosphate Region that is hydrophilic (Fig. 10a) [69]. Our results suggest that **30C** meets some features that might describe an inhibitory kinase protein molecule. **30C** might be a competitive inhibitor of ATP because it occupies the Ribose Pocket and the Binding Phosphate Region where it interacts with the G-loop. Kinase inhibitors are classified according to the nature of the inhibition. This positions the **30C** ligand in a possible type II, given that it may be ATP-competitive, induces significant conformational changes and acts on the active site [69, 70]. Figure 10b shows the noticeable conformational change induced by **30C** in the surface of the active site, modifying and exposing hydrophobic regions of this ATP-binding site to ease its molecular docking.

The **30C** ligand presents highly hydrophobic moieties. Due to this, it contacts hydrophobic residues of the binding site of *Tg*ROP18 and also can bury deeper than ATP. This enables some polar interactions to occur near the binding site: Ly281-Glu300, Gln214-Lys365, Gln216-Asp362. These interactions close the lobes and trap the **30C** molecule in the hydrophobic core of *Tg*ROP18. It has been reported that when Glu300 is bound through a salt bridge with the Lys281 residue, the N-lobe moves as a rigid structure allowing a breath-like movement, which supports the active conformation [71]. Interestingly, **30C** can bind *Tg*ROP18 preserving the Lys281-Glu300 interaction and maintaining the active conformation but without the substrate, thus, inhibiting the catalytic activity of the enzyme.

We found that the *Tg*ROP18/ATP complex, that samples around 2nd 3rd and 4th PC-mode, undergoes conformational changes that involve the C-helix, G-loop, and αN2 helix (residues 220–231). Due to their spatial disposition it is reasonable to think that these movements affect the pocket of the N-lobe where sucrose binds, was found in the crystallization. Accordingly, this relation suggests that modifications on that site by the effect of a ligand could affect the ATP-binding site by restraining these substructures.

Finally, for the possible inhibitor characteristics profile to improve the affinity and specificity of the thiazolidinone compounds for the *Tg*ROP18 protein, the interactions of ligands evaluated were analyzed and supplemented with the information collected in the literature on inhibitors types for kinase proteins. Structure-activity relationships (SAR) trends were observed when we analyzed in detail the docking conformations, the scores, and the interactions between the thiazolidinone derivatives and residues of the binding site. All compounds were recognized as potential inhibitors of studied enzymes targets within *T. gondii*. We found that hydrophobic groups attached to C2 within the thiazolidinone core facilitate the interactions of these compounds with the Adenine Pocket and Ribose Pocket regions of the active *Tg*ROP18 site through hydrophobic contacts; and also, that substitutions with aromatic rings on the secondary amine (N3) of the thiazolidinone core would have a strong tendency to interact with the Asp427 and Lys281 that play an indispensable role in the phospho-transference of the γ-phosphate group [71]. In addition, it is interesting to mention that the derivatives with higher affinity (**34A**, **36A**, **34B**, **37B** and **30C**) have a moderately bulky aromatic group attached to their hydrazine moiety, such as: naphthalene in the case of **34A**, **34B**; coumarin group in **36A**; and ferrocene group in **37B** and **30C**, which behaves similarly due to the presence of the two cyclopentadienyl rings. Based on the above, it can be suggested that fragments of this type anchored to moiety hydrazone would be modifications to be conserved on the structure of new thiazolidinone derivatives (bulky hydrophobic aromatic-hydrazone-thiazolidinone) with anti-*Toxoplasma* activity; thus **30C** derivative can be taken as a starting point for further modifications to its structure in order to design new drugs with improved affinity and specificity with promising anti-*T. gondii* activity. Further enzymatic studies are necessary to develop our knowledge of the molecular bioactivity basis of thiazolidinone derivatives.

## Conclusions

According to data published, thiazolidinone core could be considered an in vitro hit against *T. gondii*. This work presented the possible interactions of some derivatives of this core that may occur with some essential *T. gondii* proteins involved in processes as invasion, replication and proliferation. This study showed that this core is prone to interacting with the G-loop and important residues of the active site of kinase proteins of *T. gondii*. Among the evaluated derivatives, the **30C** derivative was prominent, inducing possibly, some structural changes in the *Tg*ROP18 protein, affecting some structures within the protein such as the G-loop and C-helix. It is difficult to identify the precise mechanism of action of a given compound through whole organism, especially in cases where multiple targets are hit by the same compound, as with the **30C** ligand in which three of the four protein targets achieved the lowest binding-free energies and is among the best five binding-free energies with the other protein target. In general, homologues of a drug target can also be expressed within the host; thus, resulting in adverse side effects. This is why *Tg*ROP18 could be an ideal target to inhibit, because this protein is produced exclusively by *T. gondii* and plays a major role in hiding the parasite from the immune system and becomes an important virulence factor. Therefore, this work favors a strategy that is regarded as more rational, namely target-based drug design.

## Additional files

Additional file 1: **Figure S1.** Alignment of the protein sequences of *Tg*PDI and the A chain of *h*PDI. The amino acids Phe283, Phe301, Val330, Ile335 and Ser338 of *Tg*PDI are highlighted in red. Alignment made by the Swiss-Model web server. (TIF 35295 kb)

Additional file 2: **Figure S2.** Alignment of the protein sequences of *Tg*RNR2 and the A chain of the *Pv*RNR2. The amino acids Glu172, His175, Ser176, Tyr179, Glu234, Phe238, Glu268, and His271 of *Tg*RNR2 are highlighted in red. Alignment made by the Swiss-Model web server. (TIF 35029 kb)

Additional file 3: Re-scoring file. Re-scoring results for molecular dockings performed with Autodock Vina. (TXT 4 kb)

Additional file 4: **Figure S3.** Analysis of contact frequencies in the MD runs of TgROP18/ATP and TgROP18/30C. Contact frequency between the ligand and the residues with stronger contacts. Each square represents the contact frequency of residue in a 50 ns part of one MD run. The contact frequency of the residues has been colored from red (0%) to green (100%), as shown in the palette. The contacts of TgROP18/ATP are displayed to the left; the ones of TgROP18/30C to the right. The contacts have been labeled by the sub-structure they belong to. The contacts that varied along the MD were marked with a black square. (TIFF 574 kb)

Additional file 5: **Figure S4.** Representation of the motions explained by the 1st **(a)**, 2nd **(b)**, 3rd **(c)**, and 4th **(d)** PC-mode obtained from concatenated PCA. The PC-modes are normalized to the same variance. Marks indicating hinge motion, lid-like motion, opening and rotation have been added to the figure. (TIFF 3902 kb)

## Abbreviations

AP: Adenine pocket
BPR: Binding phosphate **r**egion
CDPK: Calcium Dependent Protein Kinase
IC50: Half maximal inhibitory concentration
MM-GBSA: Molecular Mechanics/Generalized Born Surface Area
Nc: Neospora caninum
PCA: principal components analysis
PDI: Protein Disulphure Isomerase
PV: parasitophorous vacuole
RMSD: Root-Mean-Square-Deviation
RMSF: Root-Mean-Square-Fluctuation
RNR: Ribonucleotide **r**eductase
ROP: Rhoptry protein
RP: Ribose pocket
SAR: Structure-activity relationships
Tg: Toxoplasma gondii
TI: Terapeuthic Index
