# Supplementary figures and images for: Protein targets of thiazolidinone derivatives in Toxoplasma gondii and insights into their binding to ROP18
Source: BMC Genomics. 2018 Nov 29;19:856. doi: 10.1186/s12864-018-5223-7 (PMC6267824; doi:10.1186/s12864-018-5223-7)

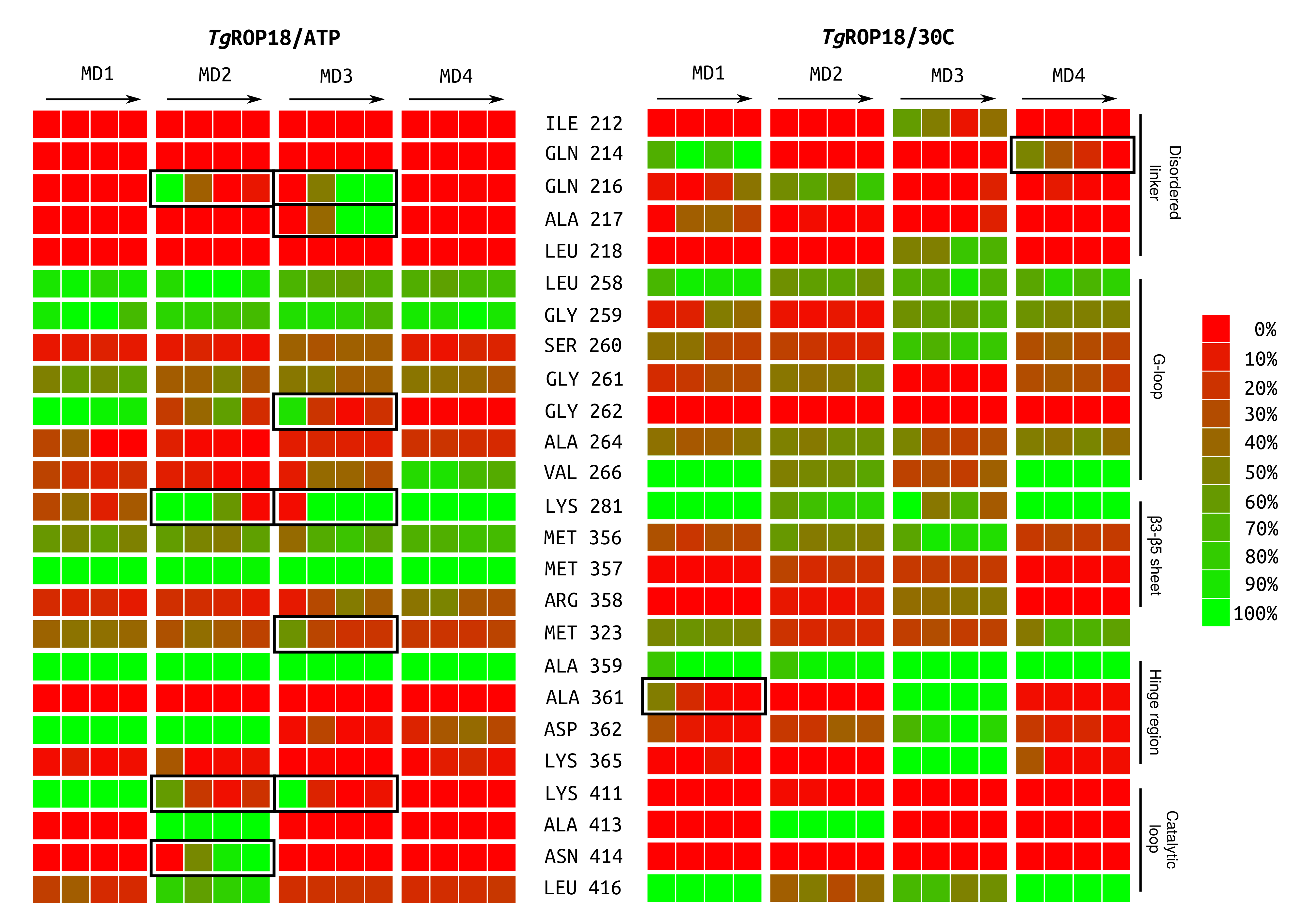

Supplement: Supplementary file 4 — Figure S3. Analysis of contact frequencies in the MD runs of TgROP18/ATP and TgROP18/30C. Contact frequency between the ligand and the residues with stronger contacts. Each square represents the contact frequency of residue in a 50 ns part of one MD run. The contact frequency of the residues has been colored from red (0%) to green (100%), as shown in the palette. The contacts of TgROP18/ATP are displayed to the left; the ones of TgROP18/30C to the right. The contacts have been labeled by the sub-structure they belong to. The contacts that varied along the MD were marked with a black square. (TIFF 574 kb) [file 12864_2018_5223_MOESM4_ESM.tiff]

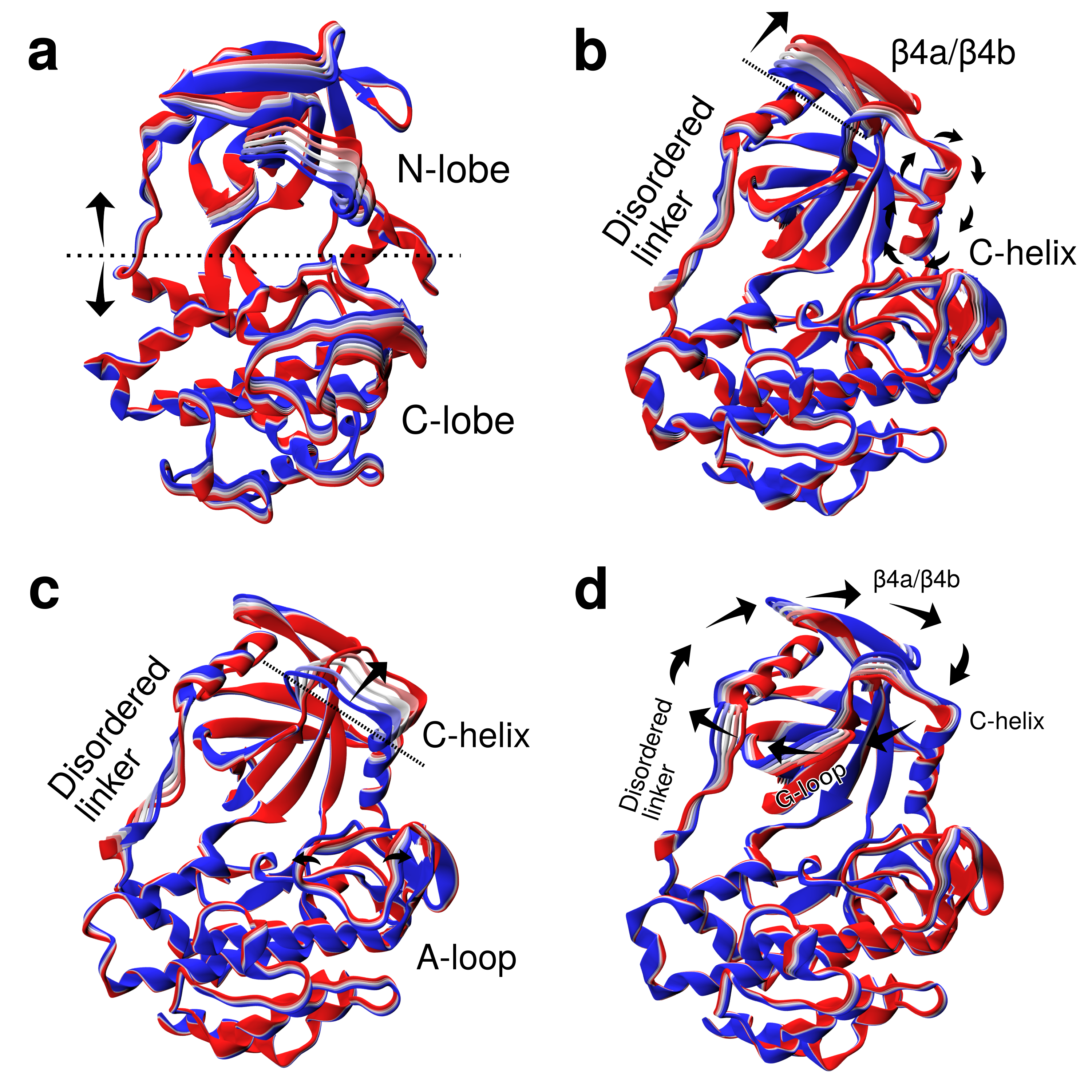

Supplement: Supplementary file 5 — Figure S4. Representation of the motions explained by the 1st (a), 2nd (b), 3rd (c), and 4th (d) PC-mode obtained from concatenated PCA. The PC-modes are normalized to the same variance. Marks indicating hinge motion, lid-like motion, opening and rotation have been added to the figure. (TIFF 3902 kb) [file 12864_2018_5223_MOESM5_ESM.tiff]
